# Mixed Views on Alcohol

**Published:** 1920-10-02

**Authors:** 


### MIXED VIEWS ON ALCOHOL.

THE subject of alcohol is interminable and apparently indeterminable. It is almost a British Medical Association tradition that at one of the breakfasts during its annual meeting the matter should be discussed in vigorous "after-breakfast" debate that discloses a sharp clash of ideas. The medical profession is divided upon it. You find a medical man—Dr. J. A. Davidson, Medical Officer to Central London District Schools—writing to the press advocating prohibition in the interests of mothers and babies, and editorial comment stating that the poison cupboard is the proper place for alcohol. There are others who just as strongly oppose prohibition, and both sides give seemingly convincing statistics to prove their contentions. Some advocate model public-houses as the best road to temperance, and they are, in turn, warned that by making public-houses too attractive they would attract more customers than the old ones, which themselves would not in those circumstances suffer diminution of custom. There are those who say you cannot make people sober by law, and those who want the State to prohibit the sale of liquor. All Scotland is at this moment agitated about the liquor question. Mr. "Pussyfoot" Johnson is back again in this country in an endeavour to extend the plan of prohibition which is by no means finally settled in the United States, and which is not, in fact, complete. But the pursuit of sobriety and the pursuit of prohibition are vastly different projects. Everyone is agreed upon the wisdom of sobriety, and controversy is confined to the means of extending it. Prohibition raises far greater issues. The average Englishman and (we may hazard the view) the average Scotsman sees no harm in the moderate consumption of intoxicating liquors. He also realises that there are special occasions, when it is important to eschew them, but in general he finds it difficult to discover why he should be forcibly prevented from drinking them at any times any more than from taking raw turnips, strong tea, or other delectable things. If he were told, in pure scientific truth, that it was an outstanding poison, it would be different; but experts are not at all agreed, and he fortifies himself by those whose views please his palate or his conscience, and is prepared to risk the consequences. How can you blame him when one generation is warned against sour milk and the next generation is prescribed it? Poisons of this kind are apparently mutable.

It is a thorny subject, on which we refrain from taking sides. But we should like to dig out for inspection an unexploded bombshell which was hurled into the *Daily Telegraph* a few days ago by its New York correspondent. The International Congress against alcoholism, which is meeting in Washington, was attended by Sir Auckland Geddes, the British Ambassador, who said England's interest in it was reflected in the fact that he had been requested by the Home Office, the Ministry of Health, and the Board of Trade to send full reports. Over twenty nations were represented, and we are told it "was the unanimous decision that the world was going dry." Reports were submitted on progress in the various countries; and the agreed estimate of the time it would take for the world to go quite dry was about fifty years.

